# Recent Advances in Bone Tissue Engineering: Enhancing the Potential of Mesenchymal Stem Cells for Regenerative Therapies

**DOI:** 10.3390/cimb47040287

**Published:** 2025-04-17

**Authors:** Milena Kostadinova, Miryana Raykovska, Radoil Simeonov, Stephan Lolov, Milena Mourdjeva

**Affiliations:** 1 Institute of Biology and Immunology of Reproduction, Department of Molecular Immunology, Bulgarian Academy of Sciences, 1113 Sofia, Bulgaria; dr_lolov@yahoo.com (S.L.); milena_mourdjeva@abv.bg (M.M.); 2 Institute of Information and Communication Technologies, Bulgarian Academy of Sciences, 1113 Sofia, Bulgaria; mirianaraykovska@gmail.com; 3 Department of Orthopedics and Traumatology, University Hospital Queen Giovanna-ISUL, 1527 Sofia, Bulgaria; radoil_shupi@abv.bg

**Keywords:** mesenchymal stem cells, critical-size bone defects, bone tissue engineering, bioprinting, osteogenic differentiation, osteoinductive factors

## Abstract

Bone tissue engineering (BTE) has emerged as a promising strategy for addressing bone defects and disorders that cannot be repaired through traditional methods. This field leverages the potential of various biomaterials, cells, and bioactive factors to promote bone regeneration. Mesenchymal stem cells (MSCs) have gained significant attention due to their osteogenic potential, which can be enhanced through osteoinductive factors. Osteoinductive factors, including growth factors like BMPs, TGF-β, VEGF, and IGF, play a crucial role in stimulating the osteodifferentiation process, thereby promoting bone regeneration. Furthermore, bioprinting technologies have opened new avenues for precisely designing scaffolds that can mimic the native bone architecture and provide a conducive environment for MSC differentiation. The integration of bioprinting with mesenchymal stem cells and osteoinductive factors has the potential to revolutionize regenerative therapies by allowing for the creation of patient-specific bone grafts. This review highlights the latest developments in MSC-based therapies, the role of osteoinductive factors, and the impact of bioprinting in advancing BTE. It also discusses future directions for improving the efficacy and clinical translation of these technologies.

## 1. Introduction

Bone is the second most transplanted tissue after blood; over two million bone grafting procedures are performed worldwide annually [1,2].

Bones are important organs that perform a wide range of functions, including structural support for the rest of the body, protection of vital internal organs and structures, maintenance of mineral homeostasis and acid–base balance, serving as a depot for growth factors and cytokines, and providing an environment for hematopoiesis in the bone marrow [3]. They mainly comprise osteocytes, osteoblasts, osteoclasts, and the extracellular matrix (ECM), which maintains a dynamic balance between bone resorption and bone formation [4,5]. Furthermore, bone is a vascularized organ with a unique capacity for self-repair after damage, although this ability is limited to small fractures with a size of a few millimeters [2]. Large bone defects in adults and children over two years of age do not reossify successfully, representing a significant biomedical problem [6].

In the case of large bone defects, treatment with various devices and implants is necessary to restore the structural functionality of the tissue. In order to support complete bone regeneration, diverse autografts, allografts, and xenografts are used [1]. Autologous bone grafts are the gold standard for successfully treating bone defects of critical dimensions. However, damage to the donor site, the limited amount of autologous grafts, and subsequent complications limit their utility. Allografts are an alternative but also present problems such as immunological rejection and the possibility of pathogen transmission [2]. Another common approach is placing non-biodegradable but mechanically stable implants, usually based on titanium, stainless steel, or ceramic [7]. Nonetheless, this approach almost always introduces the need for a secondary operative intervention to remove the implant from the body due to the occurrence of different post-surgery complications [7]. The limitations of traditional bone grafting methods were discussed recently by Hoveidaei et al. [8], all leading to the requirement of developing new approaches, including BTE and, in particular, bioprinting. At the moment, there is no unified solution, nor is there a generally accepted experimental model of large bone defects.

Several surgical specialties are related to bone defects. When discussing the means to overcome critical-size bone defects, all measures are related to three main aspects of the task, but each specialty emphasizes a different aspect. The main parameters of the “solution” are (1) appropriate shape/dimensions and mechanical strength; (2) biological tolerance through the permanent incorporation of or gradual replacement with the patient’s own tissue; (3) relation to existing or emerging infectious processes. For example, in orthopedics, the mechanical strength of the implant is of paramount importance. When the greatest problem is the presence of a cavity (like after a mastoidectomy), efforts are directed towards achieving volume by transplanting muscle and adipose tissue in addition to bone. In this regard, initial studies were aimed at replacing only the “intercellular substance”. Especially in traumatology and otosurgery, the risk of existing or emerging infection is significant. Therefore, the introduction of effective antimicrobial agents into the structure of the scaffold or even into its material composition is another important direction of scientific research.

Reconstruction in the dental and maxillofacial region presents a significant challenge because of the complex nature of the tissues and the functional demands placed upon them [9,10].

Still, orthopedics is the leading clinical discipline in terms of implant needs. The most common cause of bone damage is mechanical trauma, but other conditions are also possible, like infections, tumors, and loosening of endoprostheses of large joints. In multi-fragment fractures with avital fragments, or missing ones, bone loss is present even before the regeneration process. Bone and joint infections, whether primary or iatrogenic, are another common cause of bone loss. As a result of bacterial metabolism, which releases many exo- and endotoxins, many of which are lytic enzymes, bone resorption is observed. Tumors are undoubtedly one of the most common and severe causes of bone loss. In the radical removal of bone neoplasias, a wide resection of the affected bone is required, the replacement of which is seriously challenging. All these reasons lead to serious disability in patients and to long periods of treatment, often unsuccessful.

In addition to the problems described above, an increasing number of people suffer from osteoporosis due to the aging of the population. This reduces the quality of bone tissue and adversely affects the treatment of emerging bone damage [11], widening the group of people inquiring about bone substitution.

## 2. Bone Tissue Engineering

Personalized medicine in bone regeneration is based on an approach tailored to the specifics of the individual patient so that the grafts applied perfectly match the shape, structure, and dimensions of the defect site. In addition, cells isolated from the patient can be used and further integrated into this personalized implant, representing an autologous strategy that reduces the risk of immune rejection and inflammation [12,13].

In BTE, three key factors are required for successful bone regeneration: (1) osteoprogenitor cells, such as embryonic stem cells (ESCs), mesenchymal stem cells (MSCs), or induced pluripotent stem cells (iPSCs), which are capable of differentiating into functional bone cells; (2) specific growth factors that stimulate cell migration, proliferation, differentiation, and vascularization; and (3) biomaterials that offer a three-dimensional matrix for cell adhesion and growth.

Several methods of scaffold creation are widely used nowadays. Additive manufacturing (AM) techniques, such as fused filament fabrication (FFF) and 3D printing, have been used to generate BTE scaffolds, offering advantages in controlling scaffold structural properties such as pore size, degree of porosity, and mechanical strength [13,14]. FFF is the basic additive manufacturing process, in which the source material (a thermoformable polymer) is heated to a viscous state and extruded through a nozzle that moves along a set two-dimensional (2D) tool path, layer by layer [15]. AM techniques can be successfully implemented in customized BTE by generating a 3D computer-assisted design (CAD) model of the individual patient’s anatomical structure [14,16]. Various synthetic polymers, such as polycaprolactone (PCL), poly(lactic-co-glycolic) acid, and polyethylene glycol [17], natural polymers such as chitosan and cellulose, and also natural bone substitutes, such as calcium sulfate [2], tricalcium phosphate (TCP) [18,19], ceramics [20], bioactive glass [21], and calcium phosphate cement [22], are used in BTE and are necessary for bone regeneration to provide osteoconductive support for space filling and mechanical stability. However, their lack of/light osteoinductivity remains a challenge in their use as an alternative to natural bone [2].

Bone substitutes based on natural polymers are considered next-generation grafts. The use of β-tricalcium phosphate (β-TCP) and hydroxyapatite (HA) with biopolymers such as collagen and alginate has been studied extensively [2,23,24,25,26].

The three most commonly used mineral supplement forms of calcium phosphate [27] are TCP [28], HA crystals [29], and decellularized bone matrix (DCB) [30]. They all differ in form and function:

**TCP** contains readily available calcium and phosphates for bone production and is degraded into ions via hydrolysis and via osteoclast resorption [31,32].

**HA** is the naturally occurring crystalline form of bone mineral and is similar to TCP, except for its denser crystal structure and increased mechanical properties [27,33].

**DCB** is derived from natural (xenogeneic and allogeneic) bone sources and may include an organic protein phase such as collagen. However, a clinically available form of DCB, Bio-Oss, provides millimeter-size granules of bovine trabecular bone, with the organic phase largely removed [27,33].

An essential aspect of BTE is that the engineered and fabricated bone support is gradually degraded and replaced by new bone tissue without adverse health effects. It is important that the scaffold contributes to bone regeneration by providing a suitable environment for stem cells with osteogenic potential to differentiate and regenerate new bone. Another essential requirement for bone regeneration scaffolds is optimal vascularization. In the process of defect regeneration, blood vessels development precedes bone formation and only after functional circulation provides nutrients can new bone formation occur [34]. As mentioned above, the inflammation environment often accompanies critical-size bone defects and should be considered when designing the scaffold properties, so the inclusion of antimicrobial/antiinflammation material additives is of critical importance.

**Figure 1:**
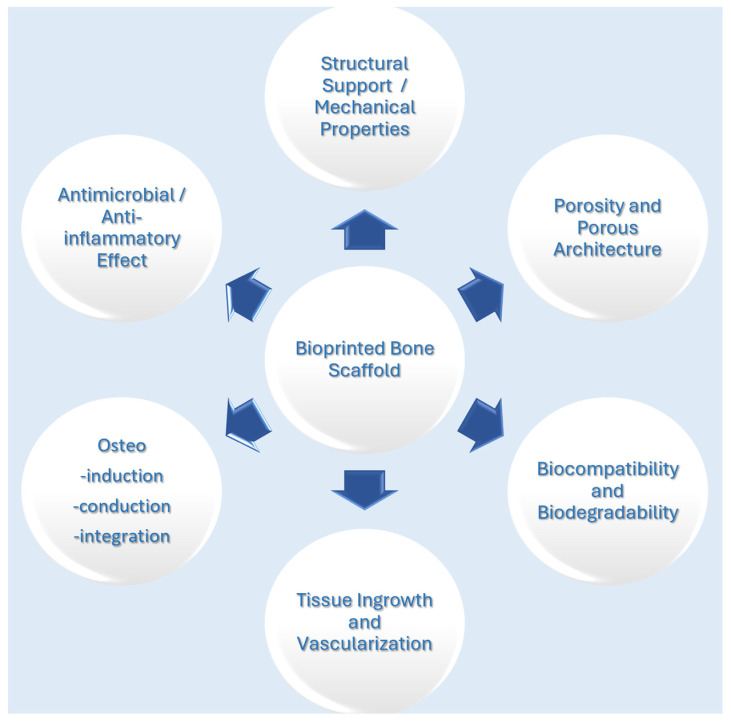
Scaffolds designed for bone regeneration must meet various requirements in terms of structural and biological properties.

Scaffolds used for tissue engineering must degrade at an appropriate rate for tissue regeneration while mechanically supporting the defect area. It is known that the regeneration of bone defects of critical dimensions requires time, and therefore, the scaffolds must have an adequate degradation rate, providing the necessary structural support to the tissue during the repair process [1,35]. On the other hand, the cells in the scaffold play an equally important role in tissue remodeling and must be able to interact with the surrounding tissue easily. Figure 1 summarizes the requirements that scaffolds designed and created for bone regeneration should meet.

As a mandatory step in the creation of a personalized bone implant, the construction of a matrix from a suitable material is relatively well studied. It should be noted that some materials have already found real clinical applications; these include polymethylmethacrylate for calvaria defects [36], PCL and β-TCP for the facial skull [37], and titanium for the femur [38]. Additional sources of information are clinical studies of application in non-osseous structures, as well as numerous animal models [39,40,41].

Bone cells are extremely sensitive to the chemical and physical properties of the scaffolds on which they are cultured. Their material composition, roughness, and topography contribute to the osteogenic process, determining cell growth and differentiation. Cell adhesion to the scaffold material represents the initial phase of cell–scaffold communication, triggering multiple cellular responses, including proliferation and differentiation, as well as vascularization [33,42].

## 3. Bioprinting

Bioprinting is a rapidly developing multidisciplinary technology, the goal of which is to produce vital three-dimensional constructs by depositing a biocompatible material (bioink) containing living cells by printing layer by layer on patient-specific digital models [8,43,44]. Bioprinting has already enabled the production of small units of tissues [45,46] and organoids [47] that share some of the functions of their native counterparts [48] and are useful as in vitro models for research and as test platforms for screening or developing drugs and personalized therapies [47]. Furthermore, 3D bioprinting has been used to produce various human tissues such as skin [49], heart [50], bone [51], liver [52], and nerve [53] tissues, as well as the extracellular matrix [54,55]. The advancement of bioprinting will allow patients to obtain access to 3D-printed tissues and organs that can replace lost or damaged ones [56].

Today, the most reported bioprinting technologies are extrusion, injected/droplet bioprinting, laser-assisted bioprinting (LAB), and stereolithography [43,57].

**Extrusion-based bioprinting** is the simplest and most widely used form, in which the bioink is ejected/expelled by a pneumatic, piston, microfluidic, or screw-based filament deposition mechanism [58]. Extrusion technology has a wide range of biocompatible materials that can be printed (e.g., cell-loaded hydrogels), but its accuracy is usually limited [59]. The viscosity and density of the bioink, the liquid phase of the bioink, the extrusion speed, and other material-specific properties, such as the ability to crosslink between the printed layers, are some of the main factors that need to be considered to achieve quality. The main disadvantages of extrusion bioprinting technology include low printable resolution, combined with possible nozzle clogging and shear stress-induced cell damage during extrusion [60].

**Injected bioprinting**, also referred to as droplet bioprinting, deposits droplets of bioink under the control of a piezoelectric or thermal system [61]. The process is performed continuously to form CAD patterns layer by layer. Droplet bioprinting offers the advantages of relatively low cost, high accuracy and speed, compatibility with various biological materials, and also the ability to deposit multiple cell types simultaneously [60]. However, it is not possible to print high cell concentrations, and low-viscosity materials reduce structural strength [62]. Another disadvantage is the need for support material, as well as possible nozzle clogging [60].

In **LAB**, cells are printed onto a receiving substrate by a pulsed laser beam at controlled speeds [63]. The receiving substrate has a ribbon structure that consists of an energy-absorbing layer on top (e.g., titanium or gold) and a bioink layer (e.g., cells and hydrogel) at the bottom. During printing, focused pulses from the laser source stimulate a region of the energy-absorbing layer. The energy vaporizes part of the donor layer to create a high-pressure bubble that propels the bioink onto the receiving substrate in the form of droplets. So, LAB is non-contact printing and avoids the problem of nozzle clogging in extrusion or droplet bioprinting, allowing for printing with higher cell densities. Furthermore, LAB does not create mechanical stress on the cells during printing, resulting in high cell viability [64]. Recent studies have demonstrated the benefits of prevascularization using LAB to promote vascularization and bone regeneration. In the work of Kérourédan et al. [65], the preorganization of human umbilical vein endothelial cells onto a newly developed biopaper using LAB led to the formation of microvascular networks. However, the printing cost is higher, and the throughput is lower [44,62,66]; there are high crosslinking requirements, and the effect of the laser on cells is still unclear [64].

**Stereolithography** is a nozzleless system that uses light to polymerize photosensitive inks in a layer-by-layer deposition process. Stereolithography is a relatively low-cost, short-time printing method and results in high resolution. Inks with high viscosity can be printed, and great cell viability can be achieved despite the possible damaging effect of UV light in photocuring. However, the reduced selection of photosensitive biomaterials limits its use [57]. Other disadvantages are the lack of multi-cell-type printability, the need for support materials, and expensive equipment [60].

Table 1 lists the most often reported bioprinting techniques in recent years, organized by major characteristics based on studies by Mobaraki et al. [44] and Sousa et al. [60].

Special attention should be paid to the macro- and microstructure of the print. The macrostructure is of particular importance for the accurate filling of the bone defect. Creating an implant with smaller dimensions implies a slightly longer healing process but easier intraoperative positioning. Generating an implant that is larger than the defect requires intraoperative fitting, probably with a better early mechanical result.

The microstructure of the bioprint is critical when it comes to parenchymal organs. Both the size and arrangement of different cell types must be taken into account. Factors such as resolution, structural fidelity, and hierarchical architectures have been extensively discussed [67,68]. Specifically for supporting tissues, and bones in particular, several factors should be considered: the correspondence between the thickness and orientation of the newly constructed lamellae (in view of the targeted mechanical resistance), the distance between them (for easier cell population and metabolism), and possibly their spatial orientation (as a prerequisite for neovascularization). The mechanisms of bone remodeling under compressive and tensile forces are relied upon to optimize construction at a later stage, while cellular self-organization during co-cultivation is utilized for the proper formation of new vessels [69].

Regardless of the method, the functioning of any of these bioprinting platforms requires bioinks with a specific set of properties, such as chemical composition, viscosity, and material rheology. In fact, the bioink is a key factor in the bioprinting strategy and is the defining element that distinguishes “bioprinting” from “3D printing”. The main advantage of bioprinting over 3D-printed cell-free scaffolds that are loaded with cells in post-printing modification procedures is that bioprints incorporate osteoinductive stem cells and osteoinductive factors, creating a microenvironment that enhances the regeneration of the bone [69].

## 4. Bioink

The success of generating functional tissues largely depends on the qualities of the bioink [43]. It must exhibit appropriate mechanical properties, stability, and biological activity to achieve and maintain the designed structures while providing a favorable environment for the cell population. The most common materials used are natural polymers [44], but there are also bioinks based on synthetic polymers [64] and hybrid/composite bioinks.

Polymeric bioinks are preferred for their low cost, biocompatibility, biodegradability, and safe processing. The main natural polymers used in bioprinting [44] are collagen [47,70], fibrin [71], silk [72], chitosan [73], alginate [74], hyaluronic acid [75], ε-polylysine [76], and gelatine methacrylate [76,77]. Water-soluble polymers, known as hydrogels, are the most used bioinks due to their chemical configuration and suitable conditions for cell growth in 3D [76,78]. Hydrogels are three-dimensional polymer networks composed primarily of proteins, peptides, and polysaccharides; they are biocompatible materials that can retain a large amount of water. As a result, they provide a favorable environment for cells [79], as they are characterized by excellent permeability for nutrients, oxygen, and other water-soluble compounds [80], have a high capacity for drug delivery, biocompatibility, and sensitivity to environmental stimuli such as temperature, pH, and solvent type [76]. Polysaccharide-based hydrogels comprise chitosan, alginates, agarose, hyaluronic acid, glucans, cellulose, guar gum, and cyclodextrin polymers. Natural polymer hydrogels offer numerous advantages, including abundant sources, porous structures, multifunctional groups, favorable swelling properties, biodegradability, and low immunogenicity [76].

In contrast, synthetic materials have various advantages over natural materials, such as the capacity to be tailored with specific physical properties and increased uniformity. The following are some examples of synthetic materials used in this field: poly (ϵ-caprolactone), poly(lactic-co-glycolic) acid, poly(l-lactic) acid, polyglycolic acid, polyurethane, polyethylene glycol, polyether ether ketone, polyvinylpyrrolidone, and Pluronic. Nevertheless, synthetic materials for 3D bioprinting have some disadvantages, such as poor biocompatibility, the potential release of toxic degradation products, and the absence of bioactive ligands [60].

Composite bioinks, also known as hybrid bioinks, are blends or multi-phase materials composed of two or more polymers with integrated bioactive inorganic additives and encapsulated cells [81]. Although polymeric materials for 3D-bioprinted structures have many advantages (low weight, low melting point, lower cost, and processing flexibility), the main problem is their low mechanical strength and functionality. To overcome this obstacle, various additives are added to the polymer matrix. One of the most used ceramic materials in tissue engineering applications is HA. Its powder is widely used in 3D printing [82]. Other used nanocomponents are β-TCP [83] and clay [84], metallic nanoparticles [85], and nano-carbon in the form of graphene [86] and carbon nanotubes [44,87].

Bioinks require crosslinking (gelling or hardening), and the shear forces resulting from this process can cause damage to the cells.

The quality of the final print product and its cell content depends on the rheological properties and crosslinking mechanisms of the bioink. During the printing process and depending on the printing technology used, several biomechanical parameters that affect the bioink must be considered for achieving the generation of an appropriate tissue architecture with good form integrity. These include flow pattern, viscosity, viscoelasticity, surface tension, flow rate, and mechanical forces such as hydrostatic pressure, shear stress, and extensional stress, which will define the printability of a biomaterial [44,88,89].

**Shear stress** is a mechanical force that occurs when fluids (e.g., bioinks) move over solid surfaces or through narrow channels [90]. In extrusion and injected bioprinting techniques, bioinks flow through the printer nozzle, where the associated shear forces can induce cellular damage [57,91]. For MSCs, excessive shear stress can lead to increased heterogeneity of cell response, induce inelastic ultra-structural distortion of the cell membrane and chromatin, and increase necrotic subpopulations post-printing [92]. The results of a study by Lemarié et al. showed that the higher the shear stress values, the lower the viable cell populations. Regarding the quantification of the fibroblasts damaged (lysis, necrosis, and apoptosis) during microextrusion, lysis was shown to be the prevailing cell death pathway compared to necrosis and apoptosis [90]. However, there is evidence that shear stress is related to stem cells’ fate and can positively influence the osteogenic differentiation of MSCs [93].

Several approaches can be used to reduce the negative impacts of shear stress on cells during bioprinting. First of all, applying low-viscosity bioinks reduces the shear stress experienced by cells during extrusion [94]. Another option is the development of shear-thinning hydrogels [57]. Shear-thinning materials are bioinks that exhibit a decrease in viscosity under shear forces and recover their original viscosity when shear stress is removed [95]. The most logical approach, but one that does not always work, is flow rate control; the careful modulation of the print head’s flow rate allows for a reduction in shear forces while maintaining adequate extrusion pressure.

Extrusion pressure is another key factor in the bioprinting process that influences MSC viability and differentiation. High extrusion pressure can cause several harmful effects, including the mechanical deformation of MSCs, leading to disrupting cell–cell and cell–matrix interactions, which may negatively affect their viability [96].

To minimize the negative effects of extrusion pressure on cell content, several strategies can be employed. By controlling the rate of extrusion pressure, it is possible to minimize the forces that affect cell survival and differentiation while maintaining the structural integrity of the printed construct [97,98]. Another commonly used technique is printing temperature control: lowering the printing temperature can help to reduce the viscosity of bioinks, allowing for printing at a lower extrusion pressure [98]. A strategy that is not so easy to implement is print head design optimization: printheads with larger nozzle diameters [99] or multi-channel nozzles [100] can help distribute the pressure more evenly, reducing the mechanical stress on the cells during extrusion.

**Crosslinking** is a process by which bioinks undergo a chemical or physical transformation to form a stable network, allowing the printed structure to hold its shape. Common crosslinking methods include chemical, physical, and thermal crosslinking [101]. Chemical crosslinking involves the use of crosslinking agents to chemically bond the polymer chains in the bioink. While chemical crosslinking stabilizes the scaffold, it can also have cytotoxic effects on cells if it is not carefully controlled [102]. Physical crosslinking is a method based on UV light exposure (photoinitiated) or ionic crosslinking (using calcium salts, for example). These methods are generally less cytotoxic but may still affect MSC viability depending on the duration and intensity of exposure [103]. Thermal crosslinking is a heat-induced gelation process, but it can cause thermal damage to sensitive cells like MSCs if temperatures exceed safe thresholds [104]. Combining different crosslinking methods, so-called dual crosslinking, can help optimize the mechanical properties of the scaffold while minimizing toxicity to MSCs [103].

The **rheology** of bioinks should be tailored to the specific printing technique and scaffold design. A shear-thinning rheology is considered an essential property for bioinks, since it performs two functions. First, the high viscosity of the material at rest ensures the mechanical stability of the prints. Second, shear-thinning qualities allow the material to be printed at a substantially lower pressure while maintaining the same printing speed, reducing the overall hydrodynamic stresses acting on cells inside the nozzles [94]. For example, the use of shear-thinning bioinks that transition from a gel-like state to a more fluid state under shear stress helps reduce the impact of extrusion forces while maintaining structural integrity after printing [57].

Bioinks commonly used in BTE are mostly hybrid since they are composed of natural or synthetic polymers, or a mix of the two, containing metallic or ceramic components, which enable the creation of structures with strong mechanical properties and simultaneously release regenerative signals to osteogenic cells to promote further regeneration and repair.

An essential factor to consider in terms of the cellular component of the bioink is the initial cell concentration. It is important to retain a sufficient number of live cells after the printing process to achieve the appropriate cell density. Bioprinting, by its mechanism, is associated with increased pressure [91], and in combination with the different crosslinking options (chemical, UV rays, etc.), it subjects the cells to shear stress. It is known that fluid shear stress or extensional flow-mediated cell deformations can damage the plasma membrane and thus lead to reduced survival [105]. This problem is even worse in the case of using hydrogels with high polymer content as the base for the bioink due to the high forces and stress on the encapsulated cells. Bioprinting of large-scale tissues or organs is even more challenging in terms of cell viability, as it needs to find a solution to maintain sufficient cell viability in the first printed layers of the construct when the printing time is more than a few hours. Cell density also affects the viscosity of the bioink—the higher the cell density, the higher the average viscosity, and this can affect the fidelity of the final construct and the functionality of the printed tissue or organ [64,106].

Despite intensive research in this rapidly developing field, data on actual bioprinting with MSCs, particularly in the area of BTE, remain very limited. More data are needed to determine the optimal combination of bioinks, cells, and osteoinductive factors, as well as the technology and parameters of printing, to achieve a sufficiently stable, durable, and functional replacement for the missing bone.

## 5. MSCs as Building Blocks for BTE

The initial selection of cells is critical for the successful fabrication of tissues and organs through bioprinting. They must have specific biological characteristics and functions so they can proliferate and differentiate into the required final cell types.

In terms of cell sources, MSCs have been widely used for bioprinting and BTE due to their unique ability to differentiate into multiple cell types, including osteoblasts, and to secrete various cytokines to regenerate damaged or injured bone tissues [107,108,109]. The majority of research has focused on bone marrow mesenchymal stem cells (BM-MSCs) due to their high osteogenic potential [110] compared to MSCs derived from other sources like adipose tissue (AT-MSCs) or perinatal tissues.

MSCs have been extensively studied in recent decades due to their functional properties. First, these cells can be induced to differentiate into any cell type depending on culture conditions and environmental cues in vitro. However, this is just one of the possible mechanisms of MSCs’ beneficial actions during repair or injury. MSCs’ biological properties put them in the focus of interest as building blocks for regenerative medicine because of their ability to migrate to the site of injury and serve as a conductor of the restoration of homeostasis, synchronizing the production of cells to be differentiated, secreting growth and angiogenic factors, and guiding the inflammatory status of the regenerating tissue, switching between a pro- and anti-inflammatory phenotype. Thus, MSCs loaded into bioinks for BTE contribute to multiple levels of bone regeneration.

For more than three decades, MSCs have been an important research interest. They were first isolated from bone marrow, and later, MSCs were shown to reside in a perivascular niche in vivo [111]. Their localization near the blood vessels explains why MSCs are nearly ubiquitous and can be isolated from most tissues—virtually all vascularized organs, including adipose tissue, articular cartilage, the brain, dental tissues, the endometrium and menstrual blood, the skin, and perinatal organs and tissues, including amniotic fluid, the amniotic membrane, the placenta, Wharton’s jelly, umbilical cord tissue, and cord blood. To identify MSCs, the International Society of Cellular Therapy postulated four minimum criteria, namely, (1) fibroblast-like morphology, (2) plastic adherence, (3) trilineage capacity for differentiation into osteoblasts, adipocytes, and chondrocytes, and (4) the expression of cell surface proteins CD73, CD90, and CD105 while lacking the expression of lineage-specific markers CD45, CD34, CD14, CD19, CD11b, and HLA-DR [112]. Although this has been the main standard for almost three decades, Pittenger et al. recently discussed the need for genome-wide gene expression studies to provide insights into the biological nature of MSCs, their expected physiological function, their role in disease pathophysiology, and their probable therapeutic mode of action [109]. These characteristics have not yet been widely introduced into the laboratory practice of groups working with MSCs, but it is good to take them into account because such studies can best characterize MSCs’ composition and function prior to their administration to patients. Promising results from clinical trials led to the FDA approval of MSC-based therapies, and ongoing investigations are exploring their potential in cartilage and bone repair, wound healing, and autoimmune disorders [113,114].

MSCs are considered the most reliable source of osteoprogenitor cells [109,115] and play a significant role in the initial formation and maintenance of bone. Endochondral ossification is a bone-healing mechanism that begins with MSCs that differentiate into chondrocytes to form cartilage, which is then calcified and subsequently remodeled into bone [116]. Intramembranous ossification is another bone regeneration mechanism involving MSCs and undifferentiated bone progenitors that directly differentiate into osteoblasts [117]. The plethora of bioactive molecules released by MSCs actively helps create an optimal regenerative microenvironment [118,119].

The specificities of in vitro osteogenic differentiation were recently summarized by Romano et al. [120], and they are as follows: (1) osteogenic induction is usually obtained in about 21 days by adding dexamethasone, β-glycerophosphate, and ascorbic acid to basic growth media; (2) specific histological staining, which reveals calcium deposits and the mineralized matrix, verifies the osteogenic phenotype (Von Kossa or Alizarin Red); (3) an increased expression of bone markers, such as Runt-related transcription factor 2 (Runx2), markers of osteoblast differentiation, such as osteonectin and osteocalcin, as well as increased synthesis of alkaline phosphatase (ALP) and collagen type I can be detected at both the mRNA or protein levels.

To elucidate the regulatory mechanism of MSC differentiation in osteoblast cells, Chen et al. [121] used single-cell multiome data to identify the regulatory elements and predict the regulation relationships between the genes and key transcription factors involved in osteogenic differentiation. The results revealed that common regulatory networks among the four donors’ MSCs were related to the stemness function of the cells, while donor-specific ones were related to their differentiation into specific cell types. The results further suggest that regulatory networks in cells with a higher potential for osteogenic differentiation may be associated with bone density-related diseases and should be considered in choosing donor MSCs for regenerative therapies. This can also be one of the explanations for the heterogeneous results reported in clinical trials with MSCs.

Last but not least, MSCs could have a significant contribution to vascularization during new bone formation. Still, one of the major challenges in BTE is the induction of angiogenesis and vasculogenesis in the implanted tissues to supply the newly formed bones with sufficient nutrients and oxygen and to withdraw the waste metabolites, thereby supporting tissue growth and maturation [8,47,122]. Transplanted MSCs can contribute to bone regeneration through angiogenesis stimulation by the expression of angiogenic factors such as VEGF, TGF-β, SDF-1, and stem cell factor (SCF) [8,123,124].

One of the most commonly used methods for angiogenesis induction in BTE is using a sustained release of angiogenic growth factors [8]. Proper dosing and kinetic management of angiogenic factors and their interaction with osteogenic processes need to be considered. Another approach is the bioprinting of stimuli-responsive biomaterials (so-called 4D printing) [125]; thus, the need to create vascular-like networks in scaffolds can be by-passed [126]. To overcome the need for a sustained production and release of growth factors, stem cells can be transfected with growth factor genes [8,120]. In some studies, the in vivo prevascularization of scaffolds by implantation into a highly perfused tissue, e.g., subcutaneous or muscular pockets, is shown [127]; thus, vessels can grow from the outside into the center of the material until complete vascularization is achieved. Recently, Kérourédan et al. reported the development of a novel biopaper able to support prevascularization organized by LAB for bone tissue engineering applications. Gelatin-based sheets incorporating bioactive glasses (BGs) were produced using various freezing methods and crosslinking parameters [65].

Co-culturing stem cells or osteoblasts with endothelial cells is a common approach to enhance vascularization [8]. Co-culture studies have demonstrated that there is crosstalk between endothelial cells and MSCs that can lead to synergistic effects on tissue regeneration [128]. In a rabbit large segmental bone defect model, the co-culture of BM-MSCs and endothelial progenitor cells (EPCs) on calcium phosphate ceramic scaffolds for better vascularization, and thus osteogenesis, was examined. Different ratios of co-cultures were first studied in vitro, and the results indicated that optimal neovascularization and osteogenesis occurred at a BM-MSC-to-EPC ratio of 1:3. Enhanced osteoid formation and bone remodeling, sustained by neovascularization, were observed compared to regular cultures. But still, suboptimal efficacy was noted compared to autologous bone grafts [129]. There are several clinical trials at different phases (I, II, or III) for bone fracture repair using BM-MSCs, AT-MSCs, umbilical cord-derived MSCs (UC-MSCs), and human amniotic epithelial cells (ClinicalTrials.gov) which were implanted either via direct injection or after seeding them onto an osteogenic matrix [123].

MSCs have been suggested to contribute to bone healing through three different mechanisms: differentiation and replacement [119], secretion of cytokines and extracellular vesicles [107,130], and immunomodulatory activity [131,132]. It is still difficult to tell which one is the leading mechanism through which MSCs enhance bone regeneration. Nevertheless, the enhancement of MSCs’ osteogenesis is directly related to improving the therapeutic effect of MSC-based BTE [4]. There is evidence that MSCs tend to differentiate into pre-osteoblasts at an intermediate stage instead of directly differentiating into osteocytes. Pre-osteoblasts develop into mature osteoblasts, which synthesize bone matrix and are then incorporated into the matrix as osteocytes [133].

## 6. Osteoinductive Factors and Their Application in BTE

Bone regeneration is a complex phenomenon that requires active signaling by numerous biomolecules at different stages of bone development [2], and signaling pathways, such as signaling through transforming growth factor β (TGF-β), Bone Morphogenic Proteins (BMPs), Wnt, and SHH, regulate the whole process. The targets of these signaling pathways are Runx2 and osterix (OSX), key transcription factors in the process of MSCs’ osteogenic differentiation [4]. Upon specific signals, the MSCs that reside in the bone marrow and periosteum differentiate into osteoblasts [134,135,136]. MSCs’ commitment to the osteoblastic phenotype is driven by a series of transcription factors in which Runx2/core-binding factor subunit alpha-1 (Runx2/Cbfa1) and the OSX are crucial for osteoblast differentiation, and the depletion of either of these two factors provokes complete damage to the mineralized skeleton [137]. When MSCs enter the pre-osteoblastic commitment pathway, a proliferative phase is activated, together with a high expression of ALP, an early osteogenic marker and critical for subsequent bone mineralization [138]. The transition to mature osteoblasts is marked by the expression of osteocalcin (OCN), bone sialoprotein (BSP), osteopontin (OPN), and collagen-I, which further contribute to the formation of the osteoid structure and mineralization [136,139].

Because of the essential role of growth factors in controlling cellular processes [140] and their ability to directly promote tissue regeneration, a wide range has been investigated and tested for therapeutic applications [141], including bone regeneration [142,143]. Factors such as fibroblast growth factor (FGF), vascular endothelial growth factor (VEGF), insulin-like growth factor (IGF), TGF-β, platelet growth factor (PDGF), and BMPs are the main participants in the process of bone regeneration [144].

**Figure 2:**
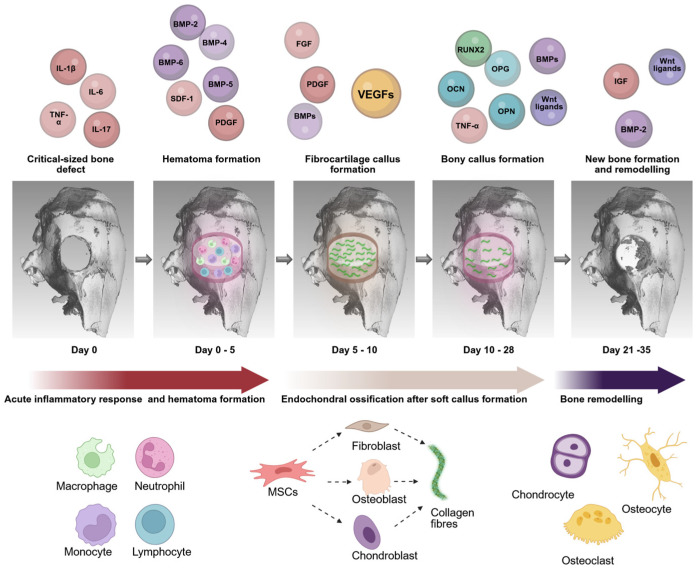
Osteoinductive factors and cytokines involved in bone tissue regeneration of a critical-sized bone defect after injury/trauma. Stages, basic processes, and cells responsible for the regeneration process.

A complex spatiotemporal cascade of cytokines orchestrates healing after bone fracture [145,146] (Figure 2). Inflammatory cytokines such as IL-1β, IL-6, IL-17, and tumor necrosis factor α (TNF-α) induce invasion by lymphocytes, plasma cells, macrophages, and osteoclasts [147]. Invading macrophages clear the necrotic centers and release TNFα, stimulating increased osteoclast activity. Osteoclasts resorb the fragmented bone mass, releasing incorporated IGF and BMPs, which induce the osteoblastic differentiation of osteoprogenitor cells or MSCs [146,148,149]. Neovascularization at the fracture site occurs early in this process as endothelial cells induce angiogenesis in response to VEGF and low oxygen concentration [146,150]. Endothelial cells are the primary source of BMPs at the fracture site, leading to the osteogenesis of osteoblasts. Osteoid production by these osteoblasts begins outside the fracture, creating a callus and mechanically integrating the bone [146]. PDGF, TGF-β and FGF, released by plasma cells, macrophages, and osteoblasts, induce and maintain cell proliferation and differentiation [149,151,152].

The inclusion of factors in bioinks is an approach to induce proliferation and differentiation in a time-dependent manner [8]. Additionally, scaffolds containing growth factors provide a better option for personalized treatment, bone defect repair, and bone regeneration in orthopedics [34]. In BTE, various approaches, such as physical entrapment, chemical binding, surface modifications, biomineralization, micro- and nanoparticle encapsulation, and genetically modified cells, are applied to provide spatiotemporal control of the delivery of a given growth factor from the scaffold [146]. Some key growth factors described above as critical during normal bone healing have been used in clinical approaches to treat bone nonunions. The timing of therapeutic growth factor delivery is crucial to optimize tissue induction while minimizing unwanted or inhibitory effects. A disadvantage of this strategy is the short half-life and high clearance rate of growth factors in vivo, especially when administered systemically [142,153]. The incorporation of growth factors into scaffolds for BTE has significant potential to improve therapeutic outcomes [2,146].

**Runx2** is a key transcription factor involved in regulating MSCs’ differentiation into osteoblasts. Runx2 increases the expression of osteoblast-specific genes and initiates mineralization [119,154]. In addition, the activation of Runx2 prevents MSCs from differentiating into other lineages, such as adipocytes [155]. Kang et al. transduced the Runx2 gene into human MSCs using a lentiviral vector and transplanted them into mice. As a result, superior bone healing was demonstrated compared to unmodified controls. The transplanted cells migrated to the fracture site and differentiated into osteoblasts to form new bone [119]. Runx2-transduced MSCs showed enhanced osteogenic properties and elevated expression of Runx2, ALP, OCN, and BSP in vivo in a model of cranial defect [156]. Runx2 may increase the efficiency of bone healing, which may be clinically relevant, but its overexpression has been associated with human malignancies in various cancers, indicating a need for caution [119].

**BMP**s, the first identified factors inducing bone regeneration in vivo, are crucial proteins for regulating the commitment of MSCs to osteoblastic fate [136]. BMPs are involved in MSCs’ differentiation into chondrocytes and osteoblasts [108], as well as in that of osteoprogenitors into osteoblasts. BMPs are produced by osteoprogenitor cells, MSCs, osteoblasts, and chondrocytes and are mainly found in the extracellular matrix of bone. BMP signaling can stimulate and play a role in almost every step of osteoblast differentiation and ultimate cell maturation [134,157]. Among BMPs, BMP-2, 4, 5, 6, 7, and 9 are necessary for bone formation, mainly by activating the Runx2, OCN, and OSX transcription factors [116,119,158], and there is evidence of an increase in their efficiency when they are heterodimeric forms of the BMP-4/-7 and BMP-2/-7 type, in vitro and in vivo [116,119]. Conversely, BMP-3 and BMP-13 are exceptions and exhibit an inhibitory function in osteoblast–osteogenic differentiation [159,160]. BMP-2 is expressed on the first day of fracture healing to stimulate MSC differentiation, and BMP-6 and -9 are expressed at later stages in animal model studies [158]. BMPs also play other roles in the healing process, such as stimulating the synthesis and secretion of other bone-related and angiogenic factors, directly activating endothelial cells for angiogenesis and regulating callus formation [119,158]. For example, BMPs have been shown to induce new bone formation at the bone defect site [142], as the process includes the initial inflammatory phase, soft callus formation, mineralization, and bone remodeling [161].

In clinical trials, recombinant human BMP-2, BMP-4, BMP-6, BMP-7, and BMP-9 have been reported to stimulate local bone regeneration by signaling MSCs’ differentiation into osteoblasts [162,163]. BMP-2 and -7 have received special attention, as they are FDA (Food and Drug Administration)-approved for bone regeneration applications [142,164]. BMP-2 is one of the most studied factors in MSCs regarding bone tissue regeneration and bioengineering; even with its short expression, it strongly positively regulates OCN expression. BMP-7, in turn, induces the expression of osteoblast differentiation markers, such as ALP, as well as collagen-I synthesis and acceleration of mineralization [165]. Meanwhile, BMP-9 has been found to strongly induce the osteogenic differentiation of MSCs by activating the canonical Wnt/β-catenin/Runx2/OCN axis, and Smad/JNK signaling induces the osteogenic differentiation of mesenchymal C3H10T1/2 cells [166,167,168]. Interestingly, BMP-9 has a synergistic effect with TGF-β in the late stages of the osteogenesis of MSCs [136,168].

BMP2-iPSC-MSCs seeded on a scaffold for BTE showed almost twice higher mineralization and ALP activity in vitro compared to the control [169].

The inclusion of BMP-4-containing nanoparticles in bioink composition had a dual effect on a rat model of cranial defect: (1) it induced the secretion of BMP-2 by M2 type macrophages; (2) BMP-4 and induced BMP-2 enhanced the osteogenic differentiation of BM-MSCs and further accelerated bone repair [170].

**TGF-β** is a potent chemotactic stimulator, enhancing the proliferation of MSCs, pre-osteoblasts, chondrocytes, and osteoblasts. TGF-β initiates signaling for BMP synthesis in osteoclast cells, inhibits osteoclast activation, and stimulates osteoclast apoptosis. TGF-β and PDGF, released by activated platelets in the early stages of fracture healing, induce the migration, activation, and proliferation of MSCs along with angiogenesis and the inflammatory response. TGF-β signaling appears activated at early stages of osteogenesis, promoting the acquisition of an immature osteoblastic phenotype by MSCs/osteoprogenitors while inhibiting further osteoblast maturation, bone mineralization, and transition to osteocytes [134,136,171]. TGF-β and BMP-2 are required for normal fracture healing. MSCs do not differentiate into the osteogenic lineage without these factors, inhibiting healing [172]. However, the osteoinductive potential of TGF-β is limited and has shown various side effects, thus limiting its clinical use for bone regeneration [119,158,172].

**Histone demethylase JMJD3** positively regulates MSC differentiation into osteoblasts in both intramembranous and endochondral bone formation. There is evidence that JMJD3 induces osteoblastic differentiation by stimulating transcription factors Runx2 and OSX and controls the expression of genes related to bone formation [119,172]. Homozygous deletion of JMJD3 strongly suppresses osteoblast differentiation and bone ossification in mice [173].

**IGF-1**, released from the bone matrix, is involved in regulating MSCs’ differentiation into osteoblasts [174] due to the activation of mTOR during the bone remodeling process [175]. On the other hand, impaired IGF-1 signaling in MSCs decreases bone formation. Knockout of IGF-1 in mouse MSCs impairs osteoblast differentiation and reduces trabecular bone formation [174].

As for **VEGF**s, in the process of bone repair, angiogenesis precedes the onset of osteogenesis. A combination of angiogenic VEGF, cell-recruiting PDGF, and osteogenic BMPs has been investigated and demonstrated a synergistic effect that is more beneficial for bone repair than either factor delivered alone [142,176].

**Cyclin-dependent kinase 1 (CDK1)** promotes the osteogenic differentiation of human MSCs through the phosphorylation of enhancer of zeste 2 polycomb repressive complex 2 subunit (EZH2). Knockdown of CDK1 with three different shRNAs blocked osteogenic differentiation and suppressed osteogenic markers, including Runx2 and OPN [177].

Regarding **Stromal cell-derived factor 1 (SDF-1)**, it has been demonstrated that SDF-1 enhances the differentiation of MSCs into osteoblasts, which is mediated by the BMP signaling pathway. Cells cultured in an osteoinductive medium with SDF-1 showed higher ALP activity than cells cultured in an osteoinductive medium alone [178]. In addition, the disruption of SDF-1 signaling decreased bone nodule mineralization and inhibited the BMP-2-induced early expression of Runx2 and OSX, which are regulators of osteogenesis. SDF-1 is upregulated at sites of injury, particularly the periosteum, and activates the CXCR4 receptor on MSCs, promoting regeneration [178]. Blocking the SDF-1/CXCR4 axis or adding SDF-1 significantly affected BMP-2-induced ALP activity and osteocalcin synthesis [179].

**Transcriptional coactivator with PDZ-binding motif (TAZ)** is a Runx2 transcriptional coactivator for the osteocalcin gene in MSCs and suppresses PPAR γ-dependent gene transcription. TAZ promotes MSC differentiation into osteoblasts and inhibits PRARγ from facilitating MSCs’ differentiation into adipocytes [180]. TAZ co-activates Runx2 in cells, which is critical for osteoblast differentiation. Experiments conducted by Hong et al. showed that the differentiation of MSCs into osteoblasts depends on Runx2 and TAZ, and their regulation may be an approach to bone formation [119].

**Fucoidan**, a sulphated polysaccharide extracted from brown seaweed, induces MSC proliferation and promotes osteoblast differentiation through JNK- and ERK-dependent BMP2-Smad 1/5/8 signaling in human MSCs. Fucoidan significantly increased ALP activity and osteocalcin and BMP-2 levels associated with bone mineralization [154]. It facilitates calcium accumulation and the regulation of osteoblast-specific genes, including ALP, Runx2, alpha-1 collagen-I, and OCN. Fucoidan also induces BMP-2 expression and stimulates the activation of extracellular signal-related kinase (ERK), c-Jun N-terminal kinase (JNK), and Smad 1/5/8 by increasing phosphorylation. Smad signaling is known to mediate the effects of BMPs, which are involved in bone formation signaling pathways. The impact of fucoidan on osteogenic differentiation was inhibited by BMP-2 knockdown and by ERK and JNK inhibitors, indicating that fucoidan affects differentiation through the BMP2-Smad 1/5/8 signaling pathway by activating ERK and JNK [119,181].

**IL-20** is involved in inhibiting osteoblast differentiation and maturation and increasing osteoclast differentiation. In vitro, IL-20 increases sclerostin and decreases OSX, Runx2, and osteoprotegerin (OPG), inhibiting osteoblast formation. IL-20 deficiency reduces fracture healing time by limiting the inhibitory effects of IL-20 on osteoblastic differentiation from MSCs and osteoprogenitor cells. IL-20 showed a significant correlation with sclerostin in patients with bone fractures and osteoporosis. Sclerostin also inhibits the differentiation, proliferation, and function of osteoblasts [138]. Anti-IL-20 monoclonal antibody 7E in a mouse model increases bone formation at the fracture site, showing its potential as a therapeutic agent for bone fractures [119,182].

The need for more understanding of MSCs’ therapeutic actions and their fate after transplantation [183,184] has made the requirements for therapeutic pre-optimization, such as optimal cell number, cell phenotype, maturity, and the mechanical properties of tissue-engineered grafts, still challenging to define [107,185,186].

Currently, bioprinting research in orthopedics is focused on the osteogenic differentiation of MSCs [62]. In 2017, Benning et al. demonstrated an enhanced proliferation and osteogenic differentiation of drop-on-demand printed MSCs in collagen and fibrin hydrogels containing hydroxyapatite [187]. Byambaa et al. developed cylindrical elements made of GelMA hydrogels enriched with VEGF and loaded with BM-MSCs and HUVECs. Bioprinted constructs have maintained cell viability and proliferation and successfully induced osteogenic differentiation [51]. Aghajanpour et al. developed a self-oxygenating bioprinted scaffold enriched with growth factors and loaded with BM-MSCs. The incorporation of both BMP-2 and calcium peroxide nanoparticles resulted in the upregulation of Runx 2, Collagen type I alpha 1, and OCN genes compared to internal references in osteogenic media [188]. In a study by Chai et al. [189], it was shown that scaffolds combining MSCs and osteoinductive factor BMP-2 had a better chance to regenerate bone defects than scaffolds with cells/factor alone. These and some other examples are summarized in Table 2.

## 7. Clinical Challenges for Bioprinted MSC Applications

The idea of BTE and 3D printing applications in the clinic dates back to the late 1990s and was initially utilized for printing dental implants, customized prostheses, and kidney bladders [88], but despite the fact that almost 40 years have passed, BTE has still not reached its dreamed aim, since only cell-free 3D printing strategies for bone repair have reached clinical application [196]. Nowadays, cell-free 3D printing is used in surgery, and orthopedic applications account for almost half of its total uses [34,197]. Three-dimensional printing technology has a wide range of development prospects in orthopedics, but other disciplines like otosurgery and dental and maxillofacial surgery are also in demand for bioprinted implants for regenerating patients’ bone defects.

Stem cell therapy presents a lot of potential for regenerative procedures in dentistry, utilizing the ability of stem cells to differentiate into different types necessary for tissue repair [198]. MSCs, particularly those derived from dental tissues such as the dental pulp, apical papilla, and periodontal ligament, are of particular interest due to their accessibility and osteogenic potential [198,199,200]. Induced pluripotent stem cells (iPSCs) also show significant potential [201]. These cells have the capability to regenerate various oral tissues, including bone, dentin, and periodontal structures, offering solutions for conditions such as tooth loss and periodontal disease [202].

Over time, 3D bioprinting has established itself as a powerful tool for creating customized scaffolds that mimic the complex architecture of dental tissues. Systematic reviews on the application of stem cells in maxillofacial regeneration have shown promising results, particularly with MSCs, though they emphasize the need for standardized protocols and long-term data [201]. Another systematic review explores the current clinical applications of stem cell therapies in facial reconstruction and regenerative surgery [203]. A novel three-dimensional construction strategy for generating mineralized bone structures using BM-MSCs is presented in [47]. In vitro and in vivo studies on bioprinted scaffold models demonstrate their potential for regenerating bone, periodontal tissue, dentin, and pulp [204]. These models often incorporate growth factors and other bioactive molecules that enhance cell differentiation and tissue formation.

The combination of dental pulp stem cell-derived exosomes and xenografts has proved to be a promising strategy for enhancing new bone formation and regenerative scores in repairing critical-size defects [205].

Stem cell therapies and 3D bioprinting are transforming the field of dentistry and offering new opportunities for the regeneration of damaged or lost tissues. Despite significant advancements, further research is required to optimize bioprinting techniques, develop the most effective bioinks, standardize stem cell protocols, and validate long-term clinical outcomes. This approach has the potential to revolutionize dental treatments and improve patient care.

## 8. Conclusions and Perspectives

Bone defects can deprive a person of the most basic support, which can lead to a range of problems. The treatment of bone defects remains a clinical challenge, and autologous transplantation is limited and cannot cover the needs. With the continuous research on bone tissue engineering, 3D-printed scaffolds have emerged; they solve the shortage of grafts and offer the option to be personally designed. Their osteogenic effect still cannot meet the needs of clinical treatment. Cells must proliferate to establish cell–cell connections and communicate with each other, as well as secrete extracellular matrix components and perform specific biological functions to integrate into the host tissue effectively [88]. Therefore, 3D-printed scaffolds loaded with growth factors were born [34].

Current technical capabilities are aimed at the independent or combined application of three new approaches: the construction of an “artificial intercellular substance” with appropriate mechanical parameters and an individualized shape (3D printing) which will overcome the shortcomings of transplantation and facilitate surgical activity; the application of different cell types, mainly MSCs, with the idea of the subsequent biodegradation of the implant and the replacement of the implant with the patient’s own tissues; and the acceleration and management of regeneration by the addition of biologically active substances. Building a structure with 3D printing is associated with many requirements for the material. In in vitro experiments, substances, whether biodegradable or not, are selected when they are suitable for the various 3D printing methods and meet certain biophysical parameters (strength, elasticity, weight, etc.). When cells are embedded into the structure during the “printing” process (so-called bioprinting), the requirements for the material become very limited, and currently, there are a relatively limited number of bioinks for “bone tissue”, mainly with collagen/gelatine [41] and cellulose microfilaments [206]. A limiting factor is also the desire for the structure to include biologically active substances, which are relatively unstable under the conditions of “printing”.

Despite intensive research, only cell-free prints are used in clinical practice as of now. In quite a lot of studies, a cell-free scaffold is printed first, and later, in a post-printing modification, it is loaded with cells. An alternative is to mix the bioink with cells, sometimes with factors as well, but this composite is injected into the site of the defect in the laboratory model, and it is not bioprinted. In the literature, plenty of osteogenic factors have been studied, some of them listed and briefly described in this review. A very limited number of them (BMP2, BMP4, BMP6, and VEGF) have already been incorporated in BTE scaffolds. Future research on osteoinductive factors may lead to the development of novel bioinks that promote bone regeneration more effectively. Future extensive research on truly bioprinted bioink/cell/factor composites is needed before their routine clinical application for bone defect repair.

## Figures and Tables

**Table 1:** Comparison of bioprinting methods in terms of their major characteristics. The “+” symbol indicates the relative performance level for each characteristic, where “++++” means excellent, and “+” means low level.

	Resolution	Speed	Mechanical Properties	Porosity	Cost
Extrusion bioprinting	+	++	++	+	++
Injected bioprinting	++	++++	+	++	+
Stereolithography	+++	+	++++	+++	+++
LAB	++++	+++	+++	++++	++++

**Table 2:** Some examples of bioprinted bone models with an added osteoinductive factor.

Cells	Bioink	Bioprinting Technique	Growth Factor	Study Type (In Vitro/In Vivo)	Bone Defect	Summary of Findings	REF
Rat BM-MSCs	GelMA/gelatine/PEG/0.4% MSNs composite hydrogels	Extrusion-based 3D bioprinting; thermo-crosslinking; photo-crosslinking of GelMA	1 μg/mL^−1^ BMP-4 added to 1 mg mL^−1^ MSNs- Mesoporous silica nanoparticles	Implanted subcutaneously on the back of C57BL/6 mouse	Calvaria defect in diabetes mellitus rats	GelMA/gelatine/PEG/MSNs composite bioinks showed satisfactory printability, mechanical stability, and biocompatibility. The sustained release of BMP-4 from MSNs induced M2-type macrophage polarization and thereby inhibited inflammatory reactions. Loading of BMP-4 and secretion of BMP-2 by M2 type macrophages promoted the osteogenic differentiation of BM-MSCs and further accelerated bone repair in DM bone defects.	[170]
Human BM-MSCs	Alginate hydrogel	Extrusion-based 3D bioprinting; ionic crosslinking	Calcium peroxide nanoparticles (CPO NPs), BMP-2 (BMP2 NPs)	In vitro	-	The viability of encapsulated hBM-MSCs was increased and osteogenic differentiation was improved. Applying a sustained-release formulation of BMP-2 resulted in greater improvement in hBM-MSCs’ osteogenic differentiation and in the upregulation of RUNX2, OCN, and COL1A1 genes.	[188]
Rat BM-MSCs	GelMA hydrogel, photo-crosslinked under UV light (365 nm) for 30 s	Injected and UV-crosslinked at the site of injury	BMP-2	SPF male SD rats	Distal femur defect, diameter 3 mm, depth of 2 mm	A photo-crosslinked BM-MSCs-BMP-2-GelMA bioactive hydrogel scaffold effectively promotes BM-MSC osteogenic differentiation and bone tissue regeneration. The active scaffold released about 70% of the BMP-2 in the first week, which continuously stimulated the adhesion and osteogenic differentiation of BM-MSCs inside and outside the scaffold.	[189]
Pre-osteoblast cell line MC3T3-E1	GelMA/HAMA hydrogel loaded with OGP	Photo-crosslinking, injectable	Osteogenic growth peptide (OGP)	In vitro	-	The hydrogel promoted cell proliferation and adhesion and increased osteogenic-related gene and protein expression in vitro.	[190]
Rat BM-MSCs	GelMA hydrogels and porous CaCO_3_ microspheres (CMs)	Injected into the site of bone defect	BMP-2	In vitro and SD rats	Skull defects	Rapid osteogenesis was induced, mainly involving MSC recruitment and differentiation in the later stage. The inflammatory response was balanced; macrophage polarization was modulated. Appropriate and timely modulation of bone healing process, such as the early inflammatory stage and the later osteogenic stage, was crucial to the healing of bone defects.	[191]
Rat BM-MSCs MC3T3-E1 cells HUVECs	Pearl powder (PP) hybrid fish gelatine methacrylate (GelMA)	Microfluidic-assisted 3D printing technology	VEGF	Rats	Skull defects	Controlled release of VEGF enables the scaffold to promote angiogenesis. A synergic effect of osteogenesis and angiogenesis was seen.	[192]
human BM-MSCs	Polypyrrole-grafted gelatin methacryloyl (GelMA-PPy) with triple crosslinking (thermo-photo-ioni-cally)	Extrusion-based 3D printing	Microcurrent stimulation (250 mV/20 min/day).	In vitro	Printed full-thickness rat bone model	Three-dimensional-bioprinted hBM-MSCs highly expressed gene hallmarks for NOTCH/mitogen-activated protein kinase (MAPK)/SMAD signaling while downregulating the Wnt/β-Catenin and epigenetic signalling pathways during osteogenic differentiation for up to 7 days.	[193]
BM-MSCs	Photo-crosslinked biomimetic methacrylated gelatin (Bio-GelMA) hydrogel	GelMA-BM-MSC suspension added into PDMS mold and photocured as scaffold	-	In vitro and rats	Segmental bone defect	The BM-MSC-carrying GelMA hydrogel scaffold has good mechanical properties and biological compatibility. It promotes the regeneration of bone and blood vessels, improves the mechanical strength of bone defects, and effectively promotes the repair of bone defects.	[194]
iPSC-derived cells via neural crest or mesoderm overexpressing BMP-6	CellInk Bone or GelXA Bone	Cellink Bio X™ 3D bioprinter (in vitro tests); In vivo bone defect was filled with spatula with ink/cell mixture and crosslinking agent was added	BMP-6	NOD/SCID mice	Cranial bone defect; frontal and parietal bones	The combination of bioprintable bioink and BMP-6 transfected iNCC-MPCs is capable of stimulating bone regeneration.	[195]
MSCs	Matrigel, fibrin, collagen, gelatine, and gelatine/alginate at various hydrogel concentrations	Drop-on-demand (DoD) printing	HA	In vitro	-	The inclusion of HA enhanced the proliferation and osteogenic differentiation of MSCs and prevented the degradation of fibrin in vitro.	[187]
MSCs HUVEC	Naturally derived hydrogel GelMA	Extrusion-based direct-writing bioprinting	Silicate nanoplatelets and VEGF	In vitro	Bone-like tissue constructs containing a perfusable vascular lumen	Encapsulated hMSCs formed a mature bone niche after 21 days of culture under the medium perfused condition.	[51]
